# Incidence and predictors of surgical site infection in Ethiopia: prospective cohort

**DOI:** 10.1186/s12879-016-2167-x

**Published:** 2017-02-03

**Authors:** Tamrat Legesse Laloto, Desta Hiko Gemeda, Sadikalmahdi Hussen Abdella

**Affiliations:** 1 Hawassa College of Health Sciences, Hawassa, Ethiopia; 2 Department of Epidemiology, Faculty of Public Health, Institute of Health Sciences, Jimma University, Jimma, Ethiopia; 3 Pharmacology and Clinical Pharmacy Department, School of Pharmacy, Addis Ababa University, Jimma, Ethiopia

**Keywords:** Antimicrobial prophylaxis, Surgical site infection, Predictors, Ethiopia

## Abstract

**Background:**

Surgical site infections are commonest nosocomial infections and responsible for considerable morbidity and mortality as well as increased hospitalizations and treatment cost related to surgical operations. The aim of this study was to determine incidence and predictors of surgical site infections at surgical ward of Hawassa University Referral Hospital, Southern Ethiopia.

**Methods:**

We performed prospective study involving 105 patients that undergone major surgical procedure at Hawassa University Referral Hospital from March 2 to May 2, 2015. Data were extracted from paper based medical charts, operational and anesthesia note, by direct observation and patients’ interview. All patients were followed daily before, during and after operation for 30 days starting from the date of operation. Data were analyzed using Statistical Package for Social Science (SPSS) for window version 20.0 software. Predictors of Surgical site infections were identified using multivariable logistic regression model. *P*-value less than 0.05 was considered to be statistically significant.

**Result:**

We studied 105 patients. Sixty four patients (61%) were males. The mean age of the patients was 30.85 ± 17.72 years. The mean Body Mass Index (BMI) was 21.6 ± 4 kg/m^2^. Twenty patients (19.1%) developed surgical site infections. Age greater than 40 years, AOR = 7.7(95% CI [1.610–40.810 *p* = 0.016,]), preoperative hospital stay more than 7 days, AOR = 22.4(95% CI [4.544–110.780, *p* = 0.001]), duration of operation more than 1 hour, AOR = 8.01(95% CI [1.562–41.099, *p* = 0.013]) and administering antimicrobial prophylaxis before 1 hour of operation, AOR = 11.1 (95% CI [1.269–75.639, *p* = 0.014]) were independent predictors for surgical site infections.

**Conclusion:**

Surgical site infection is relatively high.

## Background

Surgical site infections (SSI) are infections that occurs at or near surgical incision within 30 days of operation or after 1 year if implant is placed [1, 2]. It is the 3rd commonly reported nosocomial infection accounting for 10 to 40% of all nosocomial infections [3, 4]. Globally, SSI rates have been found to be from 2.5% to 41.9% [3, 5]. In Western countries, 2 to 5% of patients undergoing clean surgery and up to 20% of patients undergoing intra-abdominal surgery will develop SSIs [6, 7]. In Africa, surgical site infections were the leading infections in hospitals (pooled cumulative incidence of 5.6 per 100 surgical procedures), strikingly higher than proportions recorded in developed countries [8]. Another study done in Africa indicated cumulative incidence of SSIs ranged from 2.5 to 30.9% [9]. In Ethiopia, incidence rate of SSIs ranges from 10.9 to 75% [10, 11].

SSIs are preventable complications following surgery and imposes significant burden in terms of patient morbidity, mortality and increased cost of treatment. Patients who develop SSIs are up to 60% more likely to spend time in an intensive care unit, 5 times more likely to be readmitted to hospital, and 2 times more likely to die compared with patients without SSIs [7, 12–14]. It account for 3.7 million excess hospital stay days, more than $1.6 billion excess costs annually and 3.57 extra drug use [4, 13, 15–18].

Despite improvements in operating room practices, instrument sterilization methods, better surgical technique and the best efforts of infection prevention strategies, surgical site infections remain a major cause of hospital-acquired infections and rates are increasing globally even in hospitals with most modern facilities and standard protocols of preoperative preparation and antibiotic prophylaxis. In developing countries where resources are limited, even basic life-saving operations, such as appendectomies and cesarean sections, are associated with high infection rates and mortality [5, 19]. World health organization(WHO) and other studies indicated that periodic surveillance and giving feedback for surgeons on SSIs rate and associated factors can decrease up to 50% of SSIs [7, 20]. However, studies are rare in Ethiopia regarding incidence and predictors of SSI. There is no study done in Hawassa Referal Hospital regarding incidence and predictors of SSI.

Therefore, this study aimed to show the incidence rate and predictors of SSIs in surgical ward of HURH. The result of the our study will provide base line information for surgeons, governmental and non-governmental organizations working in health care system of HURH as well as in the country to decrease incidence and in the meantime complications related with SSIs.

## Methods

### Study design and participants

We conducted prospective observational study in all patients that undergone major surgical procedure at surgical ward of HURH, Southern Ethiopia from March 2 to May 2, 2015. HURH serves as a main referral center for patients living in the Southern part of Ethiopia. All patients with age ≥ 1 year admitted for elective or emergency clean and clean-contaminated surgery were included. Patients not willing to participate on the study, receiving antimicrobial during admission or stopped receiving within 48 hours before operation and patients with initial diagnosis suggestive of infection were **excluded**.

**Clean Wound** was defined as an uninfected operative wound in which no inflammation is encountered ad the respiratory, alimentary, genital or uninfected urinary tracts are not entered. In addition clean wounds are primarily closed and if necessary, drained with closed drainage. Operative incision wound that follows non-penetrating (blunt) trauma should be included in this category if they meet the criteria.

**Clean contaminated wounds** was defined as operative wounds in which respiratory, alimentary, genital or urinary tracts are entered under controlled conditions and without unusual contamination. Specifically, operations involving the biliary tract, appendix, vagina and oropharynx are included in this category provided no evidence of infection or major breaks in technique is encountered.

Data were collected by two trained Nurses (BSc) not working at surgical ward using pretested data collection tool prepared by research team.The research team supervised the data collection process daily. Socio-demographic and other patient related factors were obtained directly from patients and patient’s medical chart. Data on time of antimicrobial prophylaxis administration and intra-operative doses were collected by direct observation and from patients operation note. Data about antimicrobials administered after operation and duration of administration were extracted from patient medication chart and by direct observation. The variables included were age in years, gender, admission date, co-morbidity, body mass index, systemic steroid use, malnutrition, immunity status, ASA score, date of surgery, type of surgery (elective or emergency), wound class(clean or clean- contaminated), time of skin incision, duration of operation and amount of blood lost during operation. Regarding surgical antimicrobial prophylaxis used, place at which antimicrobials were administered (ward or operation room), generic name of antimicrobials, dose, time of first dose administration, route, time of intra-operative dose administration, time of postoperative dose given and duration of administration were recorded. We followed the patients and reviewed the charts daily before, during and after operation until the patients were discharged from the hospital and after discharge till 30 days since operation was done. Wound classification was done using Center for Disease Prevention and Control (CDC) criteria for surgical site infections surveillance [1].

### Statistical methods

Data were coded and cleaned using Epi-Data version 3.1 and exported to SPSS for window version 20.0 for analysis. Descriptive statistics were used to present socio-demographic, surgery related factors and surgical antimicrobial prophylaxis received. We used percentages, mean, standard deviation, frequencies and cross tabulation to describe patient characteristics, operational or surgical characteristics, AMP used and to determine the relationship between dependent and independent variables. In addition, bivariate and multivariable backward logistic regression with unadjusted and adjusted odd ratio was used to identify factors associated with SSIs. Variables which have a significant association at *p*-value <0.25 in the bivariate logistic regression model were fitted to multivariate logistic regression model to identify predictors of SSIs. The finding was presented using unadjusted and adjusted odds ratio and their 95% CI. *P*-value of less than 0.05 was considered as statistical significant in the multivariable analysis.

### Ethics statement

Ethical clearance was obtained from Jimma University, College of Health Sciences Ethical review board. We obtained permission from Hospital management before starting data collection. Written informed consent was obtained from each study participant before data collection. For Children less than 18 years written consent was obtained from the guardians. The Consent for both adults and children’s were documented on prepared format. Before starting the study, the protocol including the written consent was approved by Ethical review board of Jimma University. We kept the information confidential. Patients who developed SSIs were treated according to the protocol of the hospital.

## Results

### Incidence of SSIs

One –hundred twenty seven patients fulfilled the inclusion criteria, of these 22 patients were excluded based on the exclusion criteria. The analysis was done on total of 105 patients that fulfilled the inclusion and exclusion criteria. Sixty four patients (61%) were males and more than half of the patients were from rural 60(57.1%) area. The mean age of the patients was 30.85 ± 17.72 years. The mean Body Mass Index (BMI) was 21.6 ± 4 kg/m^2^, three patients (2.9%) were obese with BMI > = 30 Kg/m^2^. Five patients (4.8%) received blood transfusion preoperatively. Three (2.86%) patients received systemic steroids but none of them took for more than 3 weeks. Twenty eight (26.7%) patients were smokers. More than half of patients were under ASA score of II, 67(63.8%). The mean preoperative hospital stay and total hospital stay of the patients were 5.59 ± 7.78 and 11.03 ± 9.71 days respectively. Out of 105 patients, 20(19.1%) patients developed SSIs. Among patients who developed SSIs, 17(85%) patients developed SSIs before they were discharged from hospital (Table 1).

**Table 1:** Patient related factors at surgical ward of HURH from March 2 to May 2, 2015 (*N* = 105)

Variables	Frequency	Percent
Gender		
Female	41	39.0
Male	64	61.0
Age categories in year		
1–18	28	26.7
19–40	43	41
>40	34	32.3
Obesity		
BMI < 30 KG/m^2^	102	97.1
BMI >=30 kg/m^2^	3	2.9
Residence		
Urban	45	42.9
Rural	60	57.1
Preoperative blood transfusion		
No	100	95.2
Yes	5	4.8
Cigarette smoking		
No	77	73.3
Yes	28	26.7
ASA score		
I	29	27.6
II	67	63.8
III	9	8.6
Preoperative hospital stay		
<=7 days	78	74.3
>7 days	27	25.7

### Co-morbidities

Twelve patients (11.4%) had one or more co-morbidities namely diabetic mellitus 4(3.8%), hypertension 4(3.8%), HIV/AIDS 3(2.9%) and TB 1(0.9%).

### Types of surgical procedure

Head and neck procedure, 31(29.5%) was the leading procedure followed by gastrointestinal procedure, 30(28.6%), urologic procedure, 19(18.1%), breast procedure, 7(6.7%), hernia repair 7(6.7%) and others (Table 2).

**Table 2:** Surgical procedures done at surgical ward of HURH from March 2 to May 2, 2015 (*N* = 105)

Surgical procedures	Frequency	Percent
Head and neck surgery	31	29.5
Breast surgery	7	6.7
Gastrointestinal surgery	30	28.6
Urological surgery	19	18.1
Hepato-biliary surgery	6	5.7
Vascular procedure	4	3.8
Lipoma excision	1	1.0
Hernia repair	7	6.7
Total	105	100.0

### Surgery related factors

Sixty one (58.1%) surgical procedures were clean-contaminated and 69(65.7%) surgical procedures were elective. The mean duration of operation was 1.08 ± 0.49 and median was 1 hour. Fourteen patients (13.3%) had history of previous surgical procedure (Table 3).

**Table 3:** Surgical related factors at surgical ward of HURH from Mach 2 to May 2, 2015 (*N* = 105)

Variables	Frequency	Percent
Wound class		
Clean	44	41.9
Clean-contaminated	61	58.1
Surgery type		
Elective surgery	69	65.7
Emergency	36	34.5
Previous surgery		
Yes	14	13.3
No	91	86.7

### Predictors of surgical site infections

On bivariate logistic regression model ten variables were associated with the occurrence SSIs at *p* < 0.25. Place of residence (*p* = 0.080), age of patient greater than 40 years (*p* = 0.009), smoking (*p* = 0044), ASA score >1 (*p* = 0.067) and preoperative hospital stay more than 7 days (*p* < =001) were associated with the occurrence of SSIs. In addition, presence of one or more co-morbidities (*p* = 0.191) were associated with the occurrence of SSI (Table 4).

**Table 4:** Bivariate logistic regression model on factors associated with SSI at surgical ward of HURH from March 2 to May 2, 2015 (*N* = 105)

Variables	Surgical site infection				
	Yes N (%) 20(19.1%)	No N (%) 85(89.9%)	Crude OR	95% CI	P value
Gender					
Male	11(55%)	53(62.35%)	0.74	0.276–1.974	0.545
Female	9(45%)	32(43.52)		1	
Residence					
Rural	15(25%)	45(75%)	2.67	0.889–7.996	0.080
Urban	5(11.1%)	40(88.9)		1	
Age in year					
1–18	2(7.1%)	26(92.9%)	0.59	0.105–3.245	0.539
19–40	5(11.6%)	38(88.4%)		1	
>40	13(38.2%)	21(61.8%)	4.71	1.473–15.022	0.009
Obesity					
BMI>=30 kg/m^2^	1(33.3%)	2(66.7%)	2.18	0.188–25.354	0.532
BMI <30 kg/m^2^	19(18.6%)	83(81.4%)		1	
Preoperative blood transfusion					
Yes	1(20%)	4(80%)	1.07	0.113–10.087	0.956
No	19(19%)	81(81%)		1	
Cigarette smoking					
Yes	9(32.1%)	19(67.9%)	2.84	1.027–7.866	0.044
No	11(14.3)	66(85.7%)		1	
ASA Score					
>1	18(23.7%)	58(76.3%)	4.19	0.907–19.360	0.067
<=1	2(6.9%)	27(93.1%)		1	
Preoperative hospital stay					
>7 days	14(51.9%)	13(48.1%)	12.92	4.200–39.768	0.001
<=7 days	6(7.7%)	72(92.3%)		1	
Co-morbidity					
Present	4(33.3%)	8(66.7%)	2.41	0.646–8.967	0.191
Absent	16(17.2%)	77(82.8%)		1	
Diabetic mellitus					
Yes	1(25.0%)	3(75.0%)	1.44	0.142–14.603	0.758
No	19(18.8%)	82(81.2%)		1	
HIV/AIDS					
Yes	1(33.3%)	2(66.7%)	2.18	0.188–25.354	0.532
No	19(18.6%)	83(81.4%)		1	
Hypertension					
Yes	1(25%)	3(75%)	1.44	0.142–14.603	0.758
No	19(18.8%)	82(81.2%)		1	
Wound class					
Clean- contaminated	9(14.8%)	52(85.2%)	0.52	0.194–1.388	0.191
Clean	11(25.0%)	33(75.0%)		1	
Surgery type					
Emergency	6(16.7%)	30(83.3%)	0.79	0.274–2.256	0.654
Elective	14(20.3%)	55(79.7%)		1	
Previous surgery					
Yes	6(42.9%)	8(57.1%)	4.13	1.240–13.722	0.012
No	14(15.2%)	78(84.8)		1	
Duration of surgery					
>1 hour	15(31.9%)	32(68.1%)	4.97	1.649–14.974	0.004
<=1 hours	5(8.6%)	53(91.4%)		1	
Time of fist dose AMP administered before skin incision					
>1 hour	16(24.6%)	49(75.4%)	2.78	0.853–9.030	0.090
<=1 hour	4(10.5%)	34(89.5%)		1	
Duration of AMP administration					
>1 days	18(21.4%)	66(78.6%)	2.32	0.490–10.978	0.289
<=1 day	2(10.5%)	17(89.5%)		1	

Clean-contaminated wound class (*p* = 0.191), previous surgery (*p* = 0.012), duration of surgery more than 1 hour (*p* = 0.004) and time of first dose AMP administration 1 hour before skin incision (*p* = 0.090) were associated with incidence of SSIs (Table 4).

Age above 40 years, preoperative hospital stays more than 7 days, duration of operation more than 1 hours and administering first dose of surgical antimicrobial prophylaxis before 1 hour of skin incision were independent predictors of SSIs in multivariate logistic regression analysis (Table 5).

**Table 5:** Multivariate logistic model on predictors of SSI at surgical ward of HURH from March 2 to May 2, 2015 (N=105)

Variables	Surgical site infection				
	Yes N (%) 20(19.1%)	No N (%) 85(89.9%)	Crude OR(95% CI)	Adjusted OR(95% CI)	P
Residence					
Rural	15(25%)	45(75%)	2.67(0.889–7.996)	0.83(0.144–4.840)	0.839
Urban	5(11.1%)	40(88.9)		1	
Age in year					
1–18	2(7.1%)	26(92.9%)	0.59(0.105–3.245)	0.91(0.106–7.823)	0.933
19–40	5(11.6%)	38(88.4%)		1	
>40	13(38.2%)	21(61.8%)	4.71(1.47–15.022)	7.72(1.46–40.810)	0.016
Cigarette smoking					
Yes	9(32.1%)	19(67.9%)	2.84(1.027–7.866)	3.70(0.797–17.16)	0.095
No	11(14.3)	66(85.7%)		1	
ASA Score					
>1	18(23.7%)	58(76.3%)	4.19(0.907–19.36)	3.27(0.42–25.551)	0.258
<=1	2(6.9%)	27(93.1%)		1	
Preoperative hospital stay					
>7 days	14(51.9%)	13(48.1%)	12.92(4.20–39.77)	22.4(4.54–110.78)	0.001
<=7 days	6(7.7%)	72(92.3%)		1	
Co-morbidity					
Present	4(33.3%)	8(66.7%)	2.41(0.646–896)	1.49(0.174–12.81)	0.715
Absent	16(17.2%)	77(82.8%)		1	
Previous surgery					
Yes	6(42.9%)	8(57.1%)	4.13(1.240–13.72)	3.64(0.539–24.54)	0.185
No	14(15.2%)	78(84.8)	1	1	
Wound class					
Clean-contaminated	9(14.8%)	52(82.2%)	0.52(0.194–1.388)	0.54(0.10–2.798)	0.460
Clean	11(25.0%)	33(75.0%)		1	
Duration of surgery					
>1 hour	15(31.9%)	32(68.1%)	4.97(1.649–14.97)	8.01(1.56–41.099)	0.013
<=1 hours	5(8.6%)	53(91.4%)		1	
Time of fist dose AMP administered before skin incision					
>1 hour	16(25%)	48(75%)	2.78(0.853–9.030)	11.10(1.63–75.64)	0.014
<=1 hour	4(10.5%)	34(89.5%)		1	

Multivariable logistic regression analysis revealed patients with age greater than 40 years were 7.72 times more likely to develop SSIs compared with patients with age between 19 to 40, AOR = 7.71(95% CI [1.461–40.810]). Patients who smoke cigarette were 3.70 times more likely to develop SSIs compared with patients who didn’t smoke cigarette, AOR = 3.70(95% CI [0.797–17.165]). Moreover, patients who stay in the hospital for more than 7 days before operation were 22.23 times more likely to develop SSIs compared to patients whose operation was done within 7 days of admission AOR = 22.23(95% CI [4.544–110.781]) (Table 5).

Patients with duration of operation greater than 1 hour were 8.01 times more likely to develop SSIs compared with patients whose operation was completed within 1 hour AOR = 8.01(95% CI[1.562–41.099]). Furthermore, patients whose first dose of AMP was administered before 1 hour of skin incision were 11.10 times more likely to develop SSIs compared with patients whose first dose of AMP was administered within 1 hour AOR = 11.10(95% CI[1.629–75.639]) (Table 5).

## Discussion

Out of 105 patients who undergone surgical procedure, 20 patients developed SSIs which give overall incidence rate of 19.1%. The finding was similar to studies done in India 20.09% [21], Nigeria 20.3% [22], India 21.66% [23] and Egypt 22.6% [24]. But, lower compared to studies done in Tanzania 26% [5], India 33.5% [25] and Mekele, Ethiopia 75% [11]. The discrepancy might be due to inclusion of contaminated and dirty wound in those studies while only clean and clean-contaminated wound were included in our study. The finding was higher compared with studies done in USA 7.2% [26], France 2.5% [25], Egypt 9.2% [28] and Sudan 9% [29]. Studies done in Egypt and Sudan included only elective surgery but in our study both elective and emergency surgeries were included. This might be the reason why we find out high incidence rate of SSIs compared to studies done in both developing countries. The higher SSIs rate in present study compared to developed countries might be due modern instruments, rooms and adequate trained manpower in developed countries.

The present study revealed that patients with age greater than 40 years were 7.72 times more likely to develop SSIs compared with patients in the age range of 19 to 40 years with AOR = 7.72(95% CI[1.461–40.810,*p* = 0.016]). The result was in agreement with other studies [15, 27] confirming that as age increases the risk occurrence of SSIs increases. Different authors revealed that, as age increases the immunity will decrease and the occurrence of chronic disease that decrease the immunity of the patient, both of which synergistically predispose the patient to have SSIs [1, 10, 30].

We found that patients who smoke cigarettes were 3.7 times more likely to develop SSIs compared with patients who do not smoke cigarettes with AOR = 3.7(95% CI[0.797–17.615,*p* = 0.095]) but not independent predictor for SSIs. The finding was in line with other studies [9, 20, 22] but in studies done in Tanzania [5] and JUSH, Ethiopia [31], cigarettes smoking was found to be independent predictor for SSIs.

The sample size and observed cases with cigarettes smoking were low in our case that might be possible reason for cigarettes smoking not to be independent predictor for SSIs.

Preoperative hospital stay for longer days expose the patient for contamination or colonization by infectious pathogens which will increase risk of SSIs [1, 30]. In our study, preoperative hospital stay for more than 7 days increased the risk of SSIs by 22.44 times compared with preoperative hospital stay less than 7 days, AOR = 22.320(95% CI[4.5544–110.781,*p* = 0.001]) which was in line with other studies [24, 32]. In agreement with this finding, study done in Egypt indicated that there was no SSIs in patients whom the operation was done with 2 days of admission with statistically significant difference compared with patients whom operation was done after 2 days (*p* = 0.0034) [28]. This suggests that, shortening the preoperative hospital stay reduces the incidence rate of SSIs.

In present study, the incidence of SSIs was 25% in clean wound and 14.8% in clean-contaminated wound. The finding was consistence with other studies done in Tanzania which shows clean and clean-contaminated to be 63 and 33.7% respectively [5] and in Egypt clean and clean-contaminated wounds were 57.3 and 19.5% respectively [28]. In contrast to this, in studies done in Pure and India revealed the SSIs incidence was higher in clean-contaminated wounds, 22.8% compared to clean wounds, 15.1% [30] and in Sudan clean wound was 8% and clean-contaminated was 9.5% [27]. The low incidence of SSIs in clean-contaminated wounds in present study might be due to close precaution taken to such procedures. The other possible reason might be irrational use of Ceftiaxone for most clean wound which might resulted in resistance strains in clean wound as indicated [33].

In present study, patients who had history of surgical procedures were 3.64 times more likely to develop SSIs, AOR = 3.64(95% CI[0.539–24.537]) compared with patients who were not exposed for surgical procedure previously which was in line with other study [23]. We found prolonged operation is risk factor for SSIs because period of greatest risk for infection is the time between opening and closing the operation site [1, 30]. In addition, it increases the extent of tissue trauma because of extensive surgical procedure, stress of prolonged anesthesia, possibility of increased blood loss and also decrease the tissue level of the surgical antimicrobial prophylaxis altogether synergistically increases the risk of SSIs [5, 30].

In present study, the duration of operation greater than 1 hour increased risk of SSIs by 8.01 times compared to operation completed within 1 hour, AOR = 8.01(95% CI[1.562–41.099,*p* = 0.013]) which was in agreement with similar studies [29, 31, 34].

The administration of the first dose of surgical antimicrobial prophylaxis before 1 hour of skin incision was observed to be independent predictor for development of SSIs in previous studies [26, 35]. In agreement with this, we found administration of first dose surgical antimicrobial prophylaxis before 1 hour of skin incision increased SSIs risk by 11.10 times compared with administration of first dose of surgical antimicrobial prophylaxis within 1 hour of skin incision AOR = 11.10(95% CI[1.629–75.639,*p* = 0.014]). If the first dose of surgical antimicrobial prophylaxis administered 1 hour earlier to skin incision then the tissue as well as serum concentration of antimicrobials is not adequate enough to prevent contamination of the wound during the operation till the wound is closed. This rationale was confirmed by one study that indicated the low tissue concentration of antimicrobial at the time of wound closure was independent predictor for development of SSIs [36]. In present study, administration surgical antimicrobial prophylaxis for more than 24 hours was not found to be protective for SSIs with AOR = 2.78(95% CI 0.853–9.030). The finding was in line with other studies done in different countries [27, 37, 38]. Study done in USA showed that, administration of surgical antimicrobial prophylaxis for longer than recommended duration was independent predictor for development of antimicrobial resistance [37]. This suggests administration of surgical antimicrobial prophylaxis for more 24 hours has no additional benefits rather increasing development of antimicrobial resistance and cost.

### Limitation of the study

Our study period was short which resulted in small sample size. Variables related to health professionals, antiseptics used for patient preparation, methods used for equipment sterilization and type of anesthesia used were not included due to resource shortage.

Moreover, cases were not classified as Deep/Superficial/organ Space since these classification is not commonly practiced in the study setting.

We didn’t included CDC-NNIS risk index data in the analysis since all patients has the risk index less than 2.

Despite the limitations, our study provides vital baseline information on incidence rate and predictors of SSI in surgical ward of HURH.

## Conclusions

The overall incidence rate of SSIs was 19.1% which was lower compared with report from some developing countries and higher compared to reports from developed countries. Age greater than 40 years old, preoperative hospital stay for more than 7 days, administrating first dose surgical antimicrobial prophylaxis before 1 hour of skin incision and duration of operation for more than 1 hour were independent predictors for SSIs. Moreover administration of surgical antimicrobial prophylaxis for more than 24 hours was not protective for SSIs. Examining and identifying high risk patients and accordingly taking all appropriate care should be done to decrease the risk of SSIs. Administration of the first dose surgical antimicrobial prophylaxis within 1 hour before skin incision and shortening the preoperative hospital stay and duration operation further decrease the incidence rate of SSIs. Prolonged administration of surgical antimicrobial prophylaxis should be avoided because antimicrobial resistance is one of global threat, which results partly due to unnecessary prolonged administration. In addition, periodic surveillance on incidence rate and predictors of SSI will further decrease the incidence rate and also further researches with long study period and with large sample size should be done to get overall predictors of SSI.

## Abbreviations

AMP: Antimicrobial Prophylaxis
HURH: Hawassa University Referral Hospital
SPSS: Statistical Package for Social Sciences
SSIs: Surgical site infections
